# Social deprivation and spatial clustering of childhood asthma in Australia

**DOI:** 10.1186/s41256-024-00361-2

**Published:** 2024-06-24

**Authors:** Jahidur Rahman Khan, Raghu Lingam, Louisa Owens, Katherine Chen, Shivanthan Shanthikumar, Steve Oo, Andre Schultz, John Widger, K. Shuvo Bakar, Adam Jaffe, Nusrat Homaira

**Affiliations:** 1 School of Clinical Medicine, University of New South Wales, Randwick, NSW 2031 Australia; 2 Sydney Children’s Hospital Network, Randwick, NSW Australia; 3 Murdoch Children’s Research Institute, Melbourne, VIC Australia; 4 The Royal Children’s Hospital, Melbourne, VIC Australia; 5 Perth Children’s Hospital, Perth, WA Australia; 6 University of Western Australia, Perth, WA Australia; 7 Women’s and Children’s Hospital, Adelaide, SA Australia; 8 Sydney School of Public Health, University of Sydney, Sydney, NSW Australia; 9 James P. Grant School of Public Health, BRAC University, Dhaka, Bangladesh

**Keywords:** Childhood asthma, Spatial pattern, Social deprivation

## Abstract

**Background:**

Asthma is the most common chronic respiratory illness among children in Australia. While childhood asthma prevalence varies by region, little is known about variations at the small geographic area level. Identifying small geographic area variations in asthma is critical for highlighting hotspots for targeted interventions. This study aimed to investigate small area-level variation, spatial clustering, and sociodemographic risk factors associated with childhood asthma prevalence in Australia.

**Methods:**

Data on self-reported (by parent/carer) asthma prevalence in children aged 0–14 years at statistical area level 2 (SA2, small geographic area) and selected sociodemographic features were extracted from the national Australian Household and Population Census 2021. A spatial cluster analysis was used to detect hotspots (i.e., areas and their neighbours with higher asthma prevalence than the entire study area average) of asthma prevalence. We also used a spatial Bayesian Poisson model to examine the relationship between sociodemographic features and asthma prevalence. All analyses were performed at the SA2 level.

**Results:**

Data were analysed from 4,621,716 children aged 0–14 years from 2,321 SA2s across the whole country. Overall, children’s asthma prevalence was 6.27%, ranging from 0 to 16.5%, with significant hotspots of asthma prevalence in areas of greater socioeconomic disadvantage. Socioeconomically disadvantaged areas had significantly higher asthma prevalence than advantaged areas (prevalence ratio [PR] = 1.10, 95% credible interval [CrI] 1.06–1.14). Higher asthma prevalence was observed in areas with a higher proportion of Indigenous individuals (PR = 1.13, 95% CrI 1.10–1.17).

**Conclusions:**

We identified significant geographic variation in asthma prevalence and sociodemographic predictors associated with the variation, which may help in designing targeted asthma management strategies and considerations for service enhancement for children in socially deprived areas.

## Introduction

Globally, asthma is the most common chronic respiratory condition. In 2019, the age-standardised point prevalence and mortality rates for asthma were 3,415.5 and 5.8 per 100,000, respectively, while the age-standardised disability adjusted life year (DALY) rate was 273.6 [1]. Asthma ranks among the top 20 chronic conditions globally in terms of DALYs for children, and among the top 10 causes for school-aged children [2]. Asthma is also the most common chronic respiratory condition in Australian children, affecting an estimated 10% (approximately 460,000) of children aged 0–14 years, with a higher proportion of boys (12%) than girls (7%) being affected [3]. The hospitalisation rate for asthma in children aged 0–14 was 3.42-fold higher than that of individuals aged 15 and above, with rates of 363 and 106 per 100,000 people, respectively [4]. Asthma in children incurs substantial costs for the public healthcare system. According to a recent study, the average additional cost incurred by Medicare for asthma treatment among Australian children aged 2 to 18 is AUD190.6 million per year [5].

Asthma prevalence varies geographically throughout Australia. For example, age-standardised rates per 100 for childhood asthma ranged from 0.8 in the local government areas (LGAs) of West Daly (Northern Territory) and Hall Creek (Western Australia) to 14.2 in the LGA of Brookton (Western Australia) [6]. This spatial pattern of asthma can be attributed to local environmental features. Environments are intricate social and physical systems in which individuals reside, work, and interact, exerting various influences on people’s health status. Understanding geographical variation, identifying geographic clusters with higher asthma prevalence (i.e., hotspots), and associated local environmental features can assist policymakers in tailoring and targeting interventions to high-risk communities.

Area-level social determinants of health, also known as social deprivation or socioeconomic disadvantage, which are typically expressed as a composite index or a list of indicators, can be predictive of the spatial pattern of asthma prevalence. A high prevalence of asthma has been linked to areas with lower socioeconomic status, according to research [7]. Residing in socially deprived areas may increase asthma morbidity through differential environmental exposures, stress, and impacts on health behaviours [8]. Children’s respiratory health may be jeopardised by the adoption of unhealthy behaviours as coping mechanisms, such as smoking, by residents of socially disadvantaged neighbourhoods [8].

While there have been limited studies conducted in Australia on the markers of geographical variation in childhood asthma prevalence, previous research conducted in high-income countries has shown that area-level environmental factors play a role in the geographic variation of childhood asthma prevalence [9–12]. A study conducted in Australia’s four largest cities found a notable spatial variation in childhood asthma prevalence across small areas (i.e., statistical area level 1 (SA1)), which was explained by area-level features such as climatic factors, outdoor air pollution, and socioeconomic status [12]. A study in Chicago, USA, reported a higher asthma prevalence associated with greater neighbourhood-level (i.e., formed by aggregating census tracts) lower income but was not related to neighbourhood-level education [9]. One study in New York City, USA, reported a higher prevalence of asthma in neighbourhoods (formed by zip codes) with a greater proportion of low-income groups [13]. A study reported that criminal activity was substantially higher in neighbourhoods with a high asthma prevalence in Chicago, USA [14]. Studies from North America found that increased asthma prevalence was associated with state-level smoking rates and distance to health care facilities [11, 15].

When evaluating the geographic variation in health, it is crucial to concentrate on small area units rather than larger ones. This is because smaller areas are better at identifying localised variations, while large areas can obscure spatial heterogeneity [16]. Specifically, small geographic area analysis is needed for several paediatric asthma related policy making purposes, such as the development of relevant public health prevention programs, the distribution of resources, the creation of health policies, and the delivery of health care [17]. There is a scarcity of research in Australia that examines the variation in childhood asthma prevalence at a small area level. To address this specific gap, this study aimed to investigate small-area variation in prevalence and geographic clustering of childhood asthma and further examine its associations with sociodemographic features.

## Methods

### Study design

Our study involved an ecological analysis of data from the 2021 Australian Household and Population Census at the geographic area level. The Australian Household and Population Census collects self-reported data on a variety of indicators from all Australian individuals, families, and households. The Australian Bureau of Statistics (ABS) collected Census data, and aggregated data at various geographic area levels, which are publicly available on the ABS website (https://www.abs.gov.au/census/find-census-data/search-by-area).

### Measures

We used the Statistical Area Level 2 (SA2), a geographic area unit created by the ABS, as the analytical unit [18]. It was established to streamline the collection of data on localised statistics [18] and can reflect the underlying community structure encompassing both social and economic interactions [18]. Australia has a total of 2,473 SA2s. After excluding those with no or a small number of children aged 0–14 years (< 5), a small population size (< 200), no data on Socio-Economic Indexes for Areas (SEIFA)-Index of Relative Socio-economic Disadvantage (IRSD), and inadequate spatial connectivity with other SA2s due to being an island, this study included a total of 2,321 SA2s, accounting for 94% of SA2s. In this study, Australia is represented by 2,321 SA2s, which has a total population of 25,293,213 (mean: 10,898; range: 219 − 28,116), and 4,621,716 children aged 0–14 years (mean: 1,991; range: 10 − 7,889).

The outcome variable, at the SA2 level, was the self-reported prevalence of ever being diagnosed with asthma in children (referred to as the prevalence of self-reported asthma henceforth) aged 0–14 years. Respondents (parents or carers) were asked, ‘Has the person (named child in your household) been told by a doctor or nurse that they have asthma that has lasted or is expected to last for six months or more?’.

The two sociodemographic features analysed at SA2 level were: % of Indigenous people, and SEIFA-IRSD score and decile in Australia. The SEIFA-IRSD assesses socioeconomic disadvantage by evaluating the social and material resources accessible to locals, as well as their social participation ability [19]. In addition, the percentage of Indigenous Australians is another sociodemographic feature to consider when assessing geographic disparities in health [19, 20]. Therefore, these two features have been considered in this study. The percentage of Indigenous people at SA2 level was divided into two groups based on the median (2.08%): SA2 with a low (< 2.08%) and high (≥ 2.08%) proportion of Indigenous people. As multiple categorisations of Indigenous status would have resulted in marginal variation due to the low density of Indigenous people, we dichotomised the proportion of Indigenous people variable, which aligns with previous research [21, 22]. Lower scores or deciles of the IRSD mean more disadvantaged, and higher scores or deciles mean less disadvantaged. The SEIFA-IRSD is a validated composite index of area-level socioeconomic deprivation score based on relative disadvantage variables and developed using principal component analysis [23–25]. We divided the socioeconomic index deciles into five categories: deciles 1–2 (most disadvantaged), deciles 3–4 (disadvantaged), deciles 5–6 (medium), deciles 7–8 (advantaged), and deciles 9–10 (most advantaged).

### Statistical analysis

Descriptive statistics were computed to analyse the variable distribution in the study. Maps were used to visually display the SA2 level prevalence of asthma and socioeconomic deprivation. When working with spatial data, it is important to consider the correlation between the locations (i.e., spatial autocorrelation). The Moran’s I statistic was employed to quantify the spatial autocorrelation of variables across the study region and to support the application of spatial regression analysis [26]. The pseudo-P-value of Moran’s I was obtained through a Monte Carlo simulation with 999 replications [26]. The Queen’s contiguity spatial weights matrix was employed to establish that adjacent SA2s are considered neighbours if they have a common boundary [27].

Spatial cluster (i.e., hotspot) analysis using a univariate Local Indicator of Spatial Association (LISA) method was performed to identify spatial clusters of childhood asthma prevalence [28]. Results produce five clusters: high-high, low-low, high-low, low-high, and non-significant. High-high clusters (i.e., hot spots) are SA2s with a high asthma prevalence (i.e., higher than the average of the entire study area) surrounded by other similar SA2s, whereas low-low clusters (i.e., cold spots) are SA2s with a low asthma prevalence (i.e., lower than the average of the entire study area) surrounded by other similar SA2s. Clusters that are either high-low (i.e., SA2s with high prevalence are surrounded by SA2s with low prevalence) or low-high (i.e., SA2s with low prevalence are surrounded by SA2s with high prevalence) are known as outliers.

The distribution of sociodemographic features, such as socioeconomic disadvantage, Indigenous people, and the interaction of Indigenous people with socioeconomic disadvantage, across asthma hotspots and cold spots were examined. The association between these features and asthma clusters was assessed using the Chi-square test.

A Bayesian spatial regression model was used to estimate the association between sociodemographic features and asthma prevalence. We used multivariable spatial conditional autoregressive (CAR) Poisson regression models with different CAR priors, including Leroux, and two types of localised models (i.e., maximum number of clusters 3 and 5) to estimate the association between SA2-level asthma count and sociodemographic features, offsetting the 0-14-year-old population. We assessed model fit using the Watanabe-Akaike Information Criterion (WAIC), where a lower value signifies a better fit. A localised model that considers localised residual spatial autocorrelation with cluster 5 showed a better fit (WAIC: 18601.33) compared to the other two models (WAIC: 18756.57 for Leroux and 18734.24 for the localised model with cluster 3). The localised model partitioned areas into clusters and included a cluster-specific average model to vary the neighbourhood random effect across geographic space. For regression parameters, a Gaussian prior was used, with an Inverse Gamma (1, 0.01) prior for random effect term variability and a uniform (1, 10) prior for cluster smoothing or the penalty parameter. The study utilised Markov chain Monte Carlo (MCMC) simulations, with a total of 300,000 iterations. The initial 100,000 iterations were discarded as burn-in. The selected model showed better convergence of model parameters compared to other models, as indicated by the Geweke diagnostic. Based on the exploratory and bivariate analyses, we additionally fitted models with an interaction term that included Indigenous people and socioeconomic disadvantage. Nonetheless, the interaction term was removed from the final model due to its lack of statistical significance and no noticeable improvement in model fit. The posterior prevalence ratio (PR) and its corresponding 95% credible interval (CrI) were computed for each variable to demonstrate the magnitude of the associations. The analyses were performed using R version 4.2.1, and the CARBayes package was utilised to fit spatial models [29].

## Results

### Descriptive statistics

**Fig. 1:**
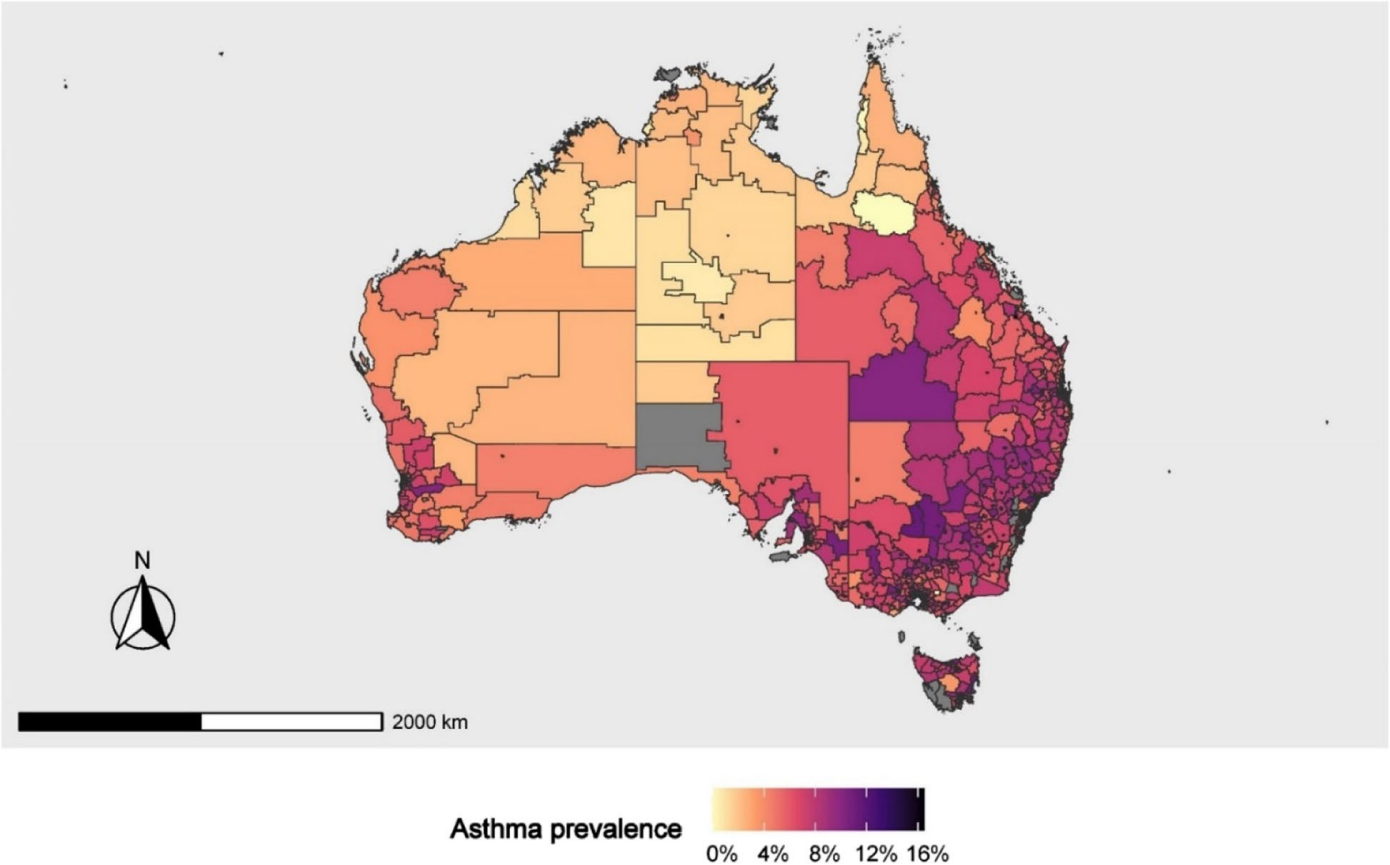
Childhood asthma prevalence map in Australia. (Note: Grey areas reflect areas with a ‘Missing’ asthma prevalence or areas that were not analysed due to exclusion criteria.)

The average prevalence of childhood asthma in Australia, based on the selected small areas (i.e., SA2s), was 6.27% (SD 1.95) (Table 1). The asthma prevalence varied across areas in Australia from 0 to 16.50%, with a low prevalence in some areas (e.g., Northern Territory – Outback, Western Australia - Outback (North), and Perth - Inner) and a high prevalence in others (e.g., Newcastle and Lake Macquarie, the Hunter Valley (excluding Newcastle), the Riverina, and the West and Northwest) (Fig. 1). The Moran’s I statistic for asthma prevalence in Australia was 0.60 (*p* < 0.001), indicating a significant spatial autocorrelation of asthma prevalence. This suggests that areas with similar asthma prevalence tend to be geographically close to each other. The average Indigenous population was 4.20% (SD 8.56%), with a socioeconomic disadvantage score of 999 (SD 82.50). The Indigenous people and socioeconomic disadvantage index score showed strong spatial autocorrelation (Moran’s I value was 0.75 and 0.60, respectively, *p* < 0.001).

**Table 1:** Descriptive statistics for the analytical dataset

Variables	*n* = 2321 SA2s	
	Average (SD)	Range
Number of 0–14 aged children	1,991 (1,227.98)	10 to 7,889
Asthma prevalence	6.27 (1.95)	0 to 16.50
Indigenous people^a^	4.20 (8.56) Median 2.08	0 to 95.90
Socioeconomic disadvantage score^b^	999 (82.50)	448 to 1,171

*SA2* Statistical area level 2, *SD* Standard deviation

^a^Proportion of people with Indigenous background

^b^Socioeconomic disadvantage was defined by the 2021 SEIFA IRSD

### Spatial clustering of asthma prevalence

**Fig. 2:**
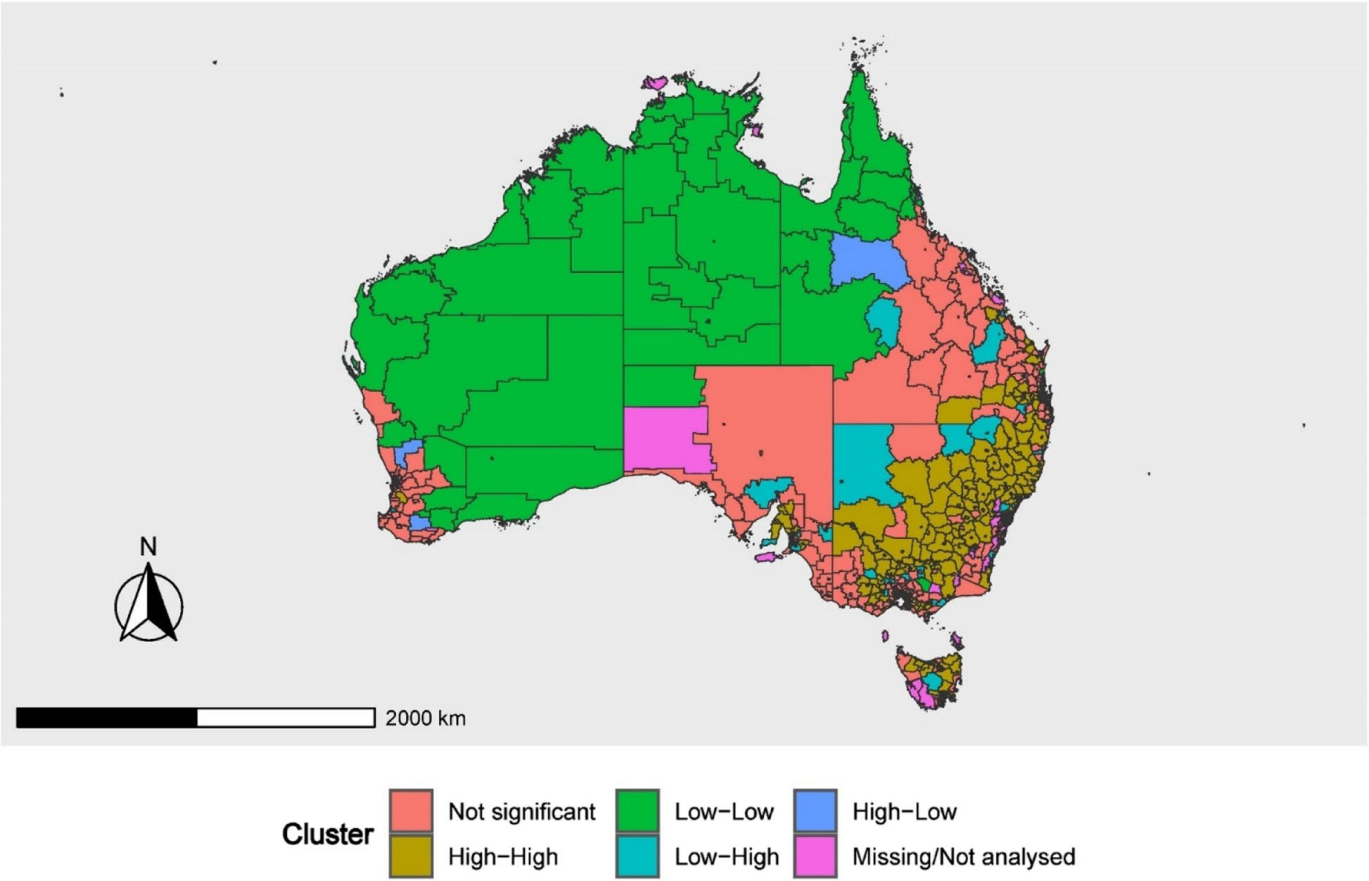
Local Indicators of Spatial Autocorrelation (LISA) map showing spatial clustering *(hotspots: High-High, cold spots: Low-Low* and outliers: *High-Low and Low-High)* of childhood asthma prevalence

The geographical cluster analysis identified 465 asthma hot spots in Australia, representing 20% of all areas, indicating areas with a statistically significant higher prevalence of childhood asthma than the overall study area average (Fig. 2). The Australian states of New South Wales, Victoria, Queensland, and Tasmania accounted for the majority of the hot spots, with 38.9%, 20.9%, 18.1%, and 11%, respectively. Asthma prevalence hotspots in New South Wales were identified in areas including Newcastle and Lake Macquarie, Hunter Valley (excluding Newcastle), Riverina and the Central Coast, and New England and the North West. Asthma hotspots in Victoria, were identified specifically in the areas of Shepparton, Ballarat, Bendigo, Geelong, and Hume. In Queensland, asthma hotspots were identified in Ipswich, Logan – Beaudesert, Wide Bay, and Moreton Bay - North. In Tasmania, hotspots were observed in the regions of Launceston and Northeast Tasmania, West Tasmania, and Northwest Tasmania. A few South Australian regions, including Barossa-Yorke-Mid North, Adelaide-North, and South, as well as the ACT regions, including Belconnen and Tuggeranong, were identified as hotspots for asthma.

### Socioeconomic disadvantage, indigenous people density and asthma prevalence

We observed a notable relationship between asthma hotspots and areas of high socioeconomic disadvantage (Table 2). In fact, more than 60% of the identified asthma hotspots were located in socioeconomically disadvantaged areas. Furthermore, 21% of the hotspots were found in areas with medium disadvantage. It is worth noting that out of the 465 small areas with asthma hotspots, 393 also exhibited a high density (i.e., ≥ 2.08%) of Indigenous people, accounting for 84.5% of all hotspot areas. When considering both Indigenous people density and socioeconomic disadvantage, approximately 57% of all hotspots were in SA2 areas with a high density of Indigenous people and high socioeconomic disadvantage.

**Table 2:** Distribution spatial clustering of childhood asthma across different sociodemographic groups

	Asthma clustering		
	Low-Low (Cold spot) *N* (%)	High-High (Hot spot) *N* (%)	*p*-value
	Total, *N* = 548	Total, *N* = 465	
**Area-level socioeconomic disadvantage**			< 0.001^a^
Most advantaged (deciles 9–10)	171 (31.2%)	24 (5.2%)	
Advantaged (deciles 7–8)	128 (23.4%)	61 (13.1%)	
Medium (deciles 5–6)	94 (17.2%)	99 (21.3%)	
Disadvantaged (deciles 3–4)	71 (13.0%)	140 (30.1%)	
Most disadvantaged (deciles 1–2)	84 (15.3%)	141 (30.3%)	
**Indigenous people (%)**			< 0.001^a^
Low (< 2.08%)	384 (70.1%)	72 (15.5%)	
High (≥ 2.08%)	164 (29.9%)	393 (84.5%)	
**Indigenous people (%) × Area-level socioeconomic disadvantage**			< 0.001^a^
Low and Most advantaged	159 (29.0%)	17 (3.7%)	
Low and Advantaged	94 (17.2%)	17 (3.7%)	
Low and Medium	61 (11.1%)	22 (4.7%)	
Low and Disadvantaged	33 (6.0%)	11 (2.4%)	
Low and Most disadvantaged	37 (6.8%)	5 (1.1%)	
High and Most advantaged	12 (2.2%)	7 (1.5%)	
High and Advantaged	34 (6.2%)	44 (9.5%)	
High and Medium	33 (6.0%)	77 (16.6%)	
High and Disadvantaged	38 (6.9%)	129 (27.7%)	
High and Most disadvantaged	47 (8.6%)	136 (29.2%)	

*N *Number of SA2s

^a^Chi-square test; Low-Low (Cold spots) - Area with a low prevalence that is surrounded by other areas that also have a low prevalence of asthma, High-High (Hot spots) - Area with a high prevalence that is surrounded by other areas that also have a high prevalence of asthma

The spatial model indicates a positive association between socioeconomic disadvantage, Indigenous people density, and asthma prevalence, as shown in Table 3. In comparison to the most advantaged areas, most disadvantaged areas had a 10% higher childhood asthma prevalence (PR = 1.10, 95% CrI 1.06–1.14), while the second most disadvantaged areas had a 4% higher prevalence (PR = 1.04, 95% CrI 1.01–1.08). The prevalence of childhood asthma was significantly higher in areas with a higher percentage of Indigenous people (PR = 1.13, 95% CrI 1.10–1.17). It is noteworthy that, despite our observation in the exploratory analysis of a higher percentage of asthma hotspots in areas with high densities of Indigenous people and socioeconomic disadvantage, the interaction effect between these two variables was not statistically significant in the spatial model and did not improve model fit; thus, this term was removed from the final model.

**Table 3:** Association between area-level sociodemographic features and childhood asthma prevalence

Variables	PR (95%CrI)
Socioeconomic disadvantage	
Most advantaged	1.00
Advantaged	0.99 (0.96–1.02)
Middle	1.01 (0.98–1.05)
Disadvantaged	1.04 (1.01–1.08)
Most disadvantaged	1.10 (1.06–1.14)
**Indigenous people (%)**	
Low (< 2.08%)	1.00
High (≥ 2.08%)	1.13 (1.10–1.17)

*PR* Prevalence ratio, *CrI *Credible interval

## Discussion

Our analysis of national data suggests that the prevalence of parent- or carer reported childhood asthma in Australia is clustered geographically. We further demonstrated that higher area-level asthma prevalence was associated with both higher area-level socioeconomic disadvantage and Indigenous people density. These findings point to potential geographic areas for focused interventions to lessen the burden of childhood asthma, with priorities set according to the sociodemographic characteristics of local populations.

Geographical clustering of childhood asthma prevalence indicates that a higher prevalence of asthma appears to cluster regionally. Substantial hot spots were mostly observed in regional or remote areas of New South Wales, Victoria, Queensland, and Tasmania. It is supported by prior research indicating that childhood asthma is more prevalent in inner-regional areas than in major cities [30]. These findings can be attributed to environmental features including socioeconomic deprivation, race or ethnicity, pollen, dust, exhaust pollutants, air pollution, violence, or crime, as well as limited access to healthcare because the majority of specialised paediatric asthma services are located in tertiary metropolitan hospitals [9–12, 31]. Research indicates that areas with low median household incomes, a high non-White population, and a high percentage of households without vehicles are associated with a high asthma hotspot [31]. Areas with high asthma prevalence had far more crime activity [14, 31]. Asthma hotspots also exhibit limited physician access [31]. There is a need for further research to assess environmental features related to geographic variation in childhood asthma in Australia. Environmental influence is better described by socioecological frameworks, which postulate that individual health and behaviour are subject to and influenced by a complex web of influences at both individual (intrapersonal) and environmental levels (interpersonal, institutional, community, and policy). The frameworks can explain the findings of this study, which assessed area-level (i.e., community-level) factors related to the spatial patterning of childhood asthma.

Our results show that areas with higher socioeconomic deprivation had a significantly higher prevalence of childhood asthma than areas with less socioeconomic deprivation, which is supported by earlier research [30]. Children residing in socially deprived areas may be more likely to have asthma due to negative environmental exposures that directly and indirectly exacerbate asthma [32]. One possible mechanism is psychosocial stress produced by local environments [32]. For example, crime is often concentrated in socioeconomically deprived areas [33], leading parents to perceive neighbourhoods as unsafe. This view may lead to children being kept indoors, increasing their exposure to allergens and harmful behaviours that can aggravate asthma [31]. Research shows that parental perceptions of neighbourhood unsafety are related to childhood asthma morbidity [34]. Other factors that might cause or intensify psychosocial stress include but are not limited to neighbourhood poverty, unemployment, substandard housing, limited access to healthcare, and greater exposure to environmental pollutants. Neighbourhoods with high levels of deprivation are typically marked by low levels of education and income, poor housing conditions, limited access to healthcare, and increased exposure to environmental pollutants, which consequently put children at higher risk of asthma [35–37]. However, we were unable to test these potential mechanisms in this study.

This study found that areas with a high proportion of Indigenous people had a higher childhood asthma prevalence. Asthma prevalence is higher among Indigenous communities in Australia [38], and previous research has reported that asthma is a significant cause of morbidity and the most prevalent chronic respiratory condition among Indigenous populations, supporting this finding [39, 40]. Various factors can contribute to the high asthma prevalence, including smoking, limited access to culturally appropriate health services, and social-environmental factors [40, 41]. For example, low income can lead to poor living conditions and exposure to environmental asthma triggers such as household moulding or air pollution, as well as limited access to healthcare resources and medications, resulting in poor asthma control and a high prevalence. According to a Western Australian study, the prevalence of asthma in Aboriginal children rises with decreasing household income [40]. Moreover, the marginalisation of Indigenous populations in disadvantaged neighbourhoods can be attributed to broader, ongoing political, economic, and social determinants of health due to the ongoing impacts of colonisation. This marginalisation often leads to increased stress levels among these populations, which in turn can contribute to higher rates of disease morbidity.

Australia has universal healthcare coverage for all citizens and eligible residents, which minimises healthcare access barriers. Research, however, suggests that environmental risk factors may contribute to the prevalence of asthma in children, even in countries with universal healthcare coverage [42]. Briefly, neighbourhood-level environmental features can contribute to neighbourhood-level variation in asthma among children by creating barriers to adequate prevention and management strategies, as well as by increasing exposure to environmental triggers for asthma. Children living in socioeconomically disadvantaged neighbourhoods have a higher likelihood of experiencing repeated visits to the emergency department due to asthma [35]. This suggests that there may be issues with asthma management, and the presence of asthma triggers. For example, managing environmental tobacco smoke and asthma triggers (e.g., dust mites) can be more challenging in public housing than in private homes, and most of the public housing is in socioeconomically deprived areas [43–45].

This study has several strengths. First, we analysed the percentage of children with asthma at a smaller geographical area level, encompassing the entirety of Australia. Secondly, the census data encompass all Australian children, unlike health surveys, which rely on a sample of the population and have limited ability to investigate small area variations. Moreover, this research employed small area spatial analysis, which allowed for a more comprehensive assessment of the spatial patterns of childhood asthma (i.e., localised variation, identifying high- or low-risk areas) and the underlying reasons, which could help public health initiatives. While our exploratory spatial analysis aids in the investigation of spatial variation in and clustering of childhood asthma, spatial regression analysis provides insight into the environmental predictors of spatial variation in childhood asthma.

This research has some limitations as well. This ecological study examines associations rather than causal relationships between environmental features and asthma prevalence. It has been suggested that parents- or carers who experience financial distress at home are more likely to report asthma morbidity in their children [46]. Therefore, the use of parents- or carer reported data for asthma in children may introduce reporting bias and may not be the most reliable method for diagnosing asthma. However, it is a widely used methodology in childhood asthma epidemiology [47]. Even though pollen, dust, climate characteristics, and household environments (e.g., smoking, allergens, mould) may contribute to asthma geographic variation, the census did not collect data on them, limiting our ability to investigate their impacts. The census was done in 2021 which coincided with the COVID-19 pandemic years. We know asthma health care utilisation was reduced in Australia during the pandemic which may have led to an estimation of a lower prevalence of asthma (the primary outcome of this analysis) [48]. However, respondents were asked if they were ever diagnosed with asthma by a physician. In addition, the pandemic presented challenges in conducting censuses. Nevertheless, by implementing effective planning and risk management strategies, such as conducting thorough testing of procedures and systems during the 2020 Census Test, the disruptions caused by the pandemic to Census field operations were mitigated, resulting in minimal impact on the accuracy and reliability of Census data [49]. In 2021, the implementation of new online self-service options aimed at enhancing the census experience gained popularity among Australian households. These options played a critical role in reducing the impact of pandemic restrictions on the distribution of census forms and other fieldwork activities [49].

This study documents significant spatial clustering of childhood asthma in Australia and highlights that prevalence of asthma is higher in some communities and in socioeconomically disadvantaged communities. This key finding can inform resource allocation and the development of strategies to minimise the inequitable burden of childhood asthma in these communities. It is crucial to employ multilevel strategies; for instance, the combined effects of environment-enhancing strategies (such as healthcare accessibility) and individual-directed interventions (such as parental counselling) are likely to be more impactful than those of either strategy alone. Through the lens of socioecological frameworks, which address and acknowledge the complex causes and consequences of health disparities, and highlight multiple levels of influence that direct the focus of health promotion programs, these strategies can be better understood. At an individual level, this could involve evidence-based proactive multi-dimensional comprehensive asthma care for children in these communities [50]. At the environmental level, this could involve initiatives to address external environmental and socioeconomic determinants of health. Overall, implementing comprehensive asthma interventions that involve children, their parents or carers, the communities they live in, and the healthcare systems can potentially enhance health outcomes for Australian children with asthma.

## Conclusions

The prevalence of childhood asthma varied across small geographical areas, with some areas having high prevalence (referred to as hot spots) and others having low prevalence (referred to as cold spots). Childhood asthma variation was found to be associated with area-level sociodemographic features, such as social deprivation and Indigenous density. The findings could potentially contribute to the development of more effective asthma management strategies and improvements in services for children living in socially deprived areas. Further research should be conducted to gain a comprehensive understanding of the spatial patterning of asthma in Australia, with a focus on environmental triggers and behavioural aspects.

## Data Availability

The study utilised publicly available datasets that can be accessed through the links provided in the Methods section.
